# Predicting attentional lapses using response time speed in continuous performance tasks

**DOI:** 10.3389/fcogn.2024.1460349

**Published:** 2024-09-04

**Authors:** Shivang Shelat, Jonathan W. Schooler, Barry Giesbrecht

**Affiliations:** ^1^Department of Psychological and Brain Sciences, University of California, Santa Barbara, Santa Barbara, CA, United States; ^2^Institute for Collaborative Biotechnologies, University of California, Santa Barbara, Santa Barbara, CA, United States

**Keywords:** vigilance, attentional lapses, mind-wandering, sustained attention, real-time monitoring

## Abstract

Failures of sustained attention, including lapses and mind-wandering, have serious consequences on cognitive task performance. In recent years, real-time triggering methods have been used to isolate periods of optimal and suboptimal attention based on patterns of response times in monotonous continuous performance tasks. In a closed-loop fashion, these triggering designs reduce the need for retrospective processing to identify periods of poor attention by using simple intrasubject response time boundaries to trigger events based on inferred attentional state. In the current review, we first discuss studies that used principal component analysis to identify response patterns that precede both task errors and phenomenological reports of mind-wandering. Then, we review designs that used real-time triggering conditions to reinforce the relationship between lapsing and memory encoding. Finally, we describe important next steps to generalize the utility of the triggering procedure across populations, validate lapse countermeasures, and shine light on the limited human capacity to maintain vigilance.

## 1 Introduction

When sustaining attention for prolonged periods, performance often fluctuates between good and bad states. Bad states are often labeled as mind-wandering or lapses (Fortenbaugh et al., 2017; Smallwood and Schooler, 2013). Lapses are studied in terms of their content (Stawarczyk et al., 2011; Banks et al., 2016), downstream effects on memory (DeBettencourt et al., 2019; Blondé et al., 2022), and in-the-moment consequences for other perceptual and cognitive phenomena (e.g., Zhang et al., 2024). Understanding attentional lapses is important in human factors and engineering psychology because of the need to reduce workplace risks through validated countermeasures (e.g., Yanko and Spalek, 2014).

Sustained attention lapses are often studied using continuous performance tasks (CPTs), which require subjects to respond to a rare target (e.g., Psychomotor Vigilance Task; Dinges and Powell, 1985). However, because the design of typical CPTs requires rare responses to infrequent target events, the resulting behavioral indices only provide an intermittent, non-continuous, measurement of state and cannot track states prior to critical events, such as errors. A new class of CPTs that requires responses on all (e.g., ANTI-Vea; Luna et al., 2018) or most (e.g., gradCPT; Esterman et al., 2013) trials instead allows for finer-grained measurement of small-scale fluctuations in response patterns over time. A well-established exemplar of these tasks is the Sustained Attention to Response Task (SART; Robertson et al., 1997), which requires monotonous responding (i.e., keypresses) to frequent non-target stimuli. At rare moments, an infrequent target is presented and it requires either no response or a different keypress. Participants inhibit their prepotent response and choose the correct response. Errors on infrequent trials are considered to be a behavioral marker of lapsing attention, impulsive responding, or a strategy that favors speed over accuracy (McVay and Kane, 2009; Robertson et al., 1997; Seli et al., 2013; Helton, 2009). Additionally, high response time (RT) variability indicates a state of poor attentional control and has been linked to trait-level attention deficits (Castellanos et al., 2006; Vaurio et al., 2009).

These types of CPTs are a valuable methodological tool because they require continual responding over the course of the task. RTs for both frequent and infrequent trials provide insight into the participant's cognitive state as they fluctuate between optimal and suboptimal responding (Esterman et al., 2013). Fluctuations can be analyzed using *post-hoc* methods that look back at periods of responding and characterize them as “in the zone” or “zoning out” based on RT variability. Recent work shows that clinically-relevant individual difference metrics such as one's maximal attention span can be derived from this retrospective approach (Simon et al., 2023). However, in the past few years, RT-based real-time triggering procedures that are adaptive to individual subjects and do not require *post-hoc* methods to delineate attentional states have successfully anticipated lapsing attention in a closed-loop fashion. Additionally, these methods can predict lapses without neurophysiological instruments and can inform the design of tasks that test relationships between attentional states and other psychological phenomena.

In this mini-review, we first discuss CPT studies that retrospectively assess RT patterns that coincide with particular attentional states. We then review papers using real-time, closed-loop RT tracking methodologies to provide convergent evidence for a relationship between lapsing attention, phenomenological state, and impaired memory encoding. Finally, we discuss the importance of testing the sensitivity of individual differences to real-time triggering designs and evaluating attentional lapse countermeasures. We preface this review by noting that a state of mind-wandering may not necessarily be the same as an attentional failure. Moments of “bad attention” may not always mean that one is engaged in task-unrelated thinking (Van den Driessche et al., 2017). However, since both mind-wandering and attentional failures implicate goal-oriented behavior and task performance, we use the umbrella term “lapse.” Discriminating mind-wandering from attentional failures is an important future direction, but it is beyond the present scope (Unsworth et al., 2021).

## 2 Response time patterns coincident with changes in attentional state

Patterns of fast responding precede errors on the SART (Larue et al., 2010). Temporally contiguous trials (i.e., windows) with relatively fast RTs may represent an inattentive, unaware state induced by monotonous responding. However, it is unclear whether RTs are reliable predictors of the subjective experience of mind-wandering or other consequences of lapsing. Smallwood et al. (2008b) administered the SART to a small sample of undergraduate students (*n* = 22) and used principal components analysis (PCA) to identify different patterns of responding preceding both errors and self-reported task-unrelated thoughts (TUTs). TUTs are a common operationalization of mind-wandering in the literature (Seli et al., 2018a,b; but see Christoff et al., 2018). Smallwood et al. defined TUTs with awareness as “tune outs” and those without awareness as “zone outs,” where the latter is more detrimental to performance than the former (Schooler et al., 2011; Smallwood et al., 2007). Of the three factors that emerged from their analysis, accelerating responses (speeding) seemed to predict both errors and zone outs (see also Marcusson-Clavertz et al., 2012). This suggests that fast RT windows are a promising unobtrusive marker of suboptimal attention, specifically a state of task-unrelated thinking without awareness.

Two limitations of Smallwood et al. (2008b)'s study were its small sample size and the use of unstandardized within-participant RTs. To address these limitations, McVay and Kane (2012) conducted a retrospective analysis on SART data from a larger amalgamated dataset (*n* = 386) to test whether speeding RTs could serve as an objective marker of mind-wandering. Speeding predicted errors on the SART, but did not consistently predict TUTs. Especially slow RTs compared to intrasubject RT averages predicted on-task reports, suggesting that slow, controlled responding could be a valid RT marker for a focused state. Their thought sampling procedure, however, did not gauge TUT awareness. Instead, participants classified their thoughts into discrete categories: the task, task performance, everyday stuff, current state of being, personal worries, daydreams, or other. The first option was classified as on-task, the second as task-related interference, and all others as TUTs (Kane et al., 2021). These results suggest that slow RT windows may represent a state of good attention, but do not corroborate prior findings that fast windows reflect mind-wandering.

McVay and Kane (2012) results may have diverged from Smallwood et al. (2008b) because they did not examine the thought quality of awareness. More recent work has addressed this issue by administering the SART with intermittent probes with 6-point Likert scales indexing task-relatedness and awareness of thoughts (Polychroni et al., 2020). The observed RT patterns did not predict attentional state, although slowing predicted less awareness of on-task states and an oscillation from fast-to-slow-to-fast responding predicted less awareness of TUTs.

In summary, the investigation into response time patterns and their relationship with attentional states has yielded mixed results. Initial findings suggested that fast response times could serve as an indicator of mind-wandering, specifically when participants were unaware of their thoughts; however, subsequent research failed to replicate this pattern.

## 3 Real-time triggering designs with response times

Instead of the *post-hoc* approach on data from whole samples, custom procedures that incorporate moment-by-moment RT fluctuations within each individual may be a more viable technique to capture shifts in attentional states and mind-wandering episodes. Researchers have designed simple tasks that calculate patterns in RT in real-time to trigger events that assess phenomenological or behavioral markers of lapsing attention. In this section, we discuss four empirical articles that used triggering procedures in CPTs to successfully foresee lapses. These methods usually rely on simple mathematical formulas that can be executed on a trial-by-trial basis in real time.

One early triggering study modified a reading task to mimic a CPT by requiring participants to press the spacebar after reading each word from a passage (Franklin et al., 2011). The presentation of thought probes was triggered when participants were going relatively fast (lapsing) or slow (attentive). Fast triggers occurred when a preceding ten-trial RT window average dipped below 55% of the cumulative average, and slow triggers occurred when the ten-trial RT average was between 130 and 175% of the cumulative average. This scheme was advantageous because it used custom, intrasubject bounds to calculate and update triggering conditions during the task. The results showed that the algorithm successfully anticipated episodes of mind-wandering and also predicted poorer performance on a reading comprehension posttest. As a subsequent step, RT in a non-probed sample was examined to infer mind-wandering rates based on their algorithm's predictions. Fast RT windows predicted worse test performance, validating the procedure's utility in circumventing the need for attentional state self-reports. This study serves as the first closed-loop paradigm that used RT in a CPT to predict both reportable TUTs and impairment in memory encoding, which is a widely documented consequence of lapsing attention (Mooneyham and Schooler, 2013; Blondé et al., 2022).

Where Franklin et al. (2011) used the closed-loop approach within the context of a reading task, Henríquez et al. (2016) used a real-time triggering procedure within a SART. The target/non-target discrimination in the SART is typically easy and the monotony of the task induces periods of inattention. Thought probes were triggered whenever a single RT exceeded two standard deviations from the mean of the participant's last five RTs. Participants were then prompted to report whether they were focused on the task or not. The findings revealed that extreme slowing between the fourth and fifth RT in the preceding window predicted TUTs. While this generally deviates from the RT window approach proposed by other studies reviewed here (i.e., Smallwood et al., 2008b; Franklin et al., 2011), it highlights the potential of using variability within a narrow RT window to anticipate mind-wandering (Jalava and Wammes, 2024).

Studying the phenomenological experience of mind-wandering via thought sampling has been fruitful for identifying antecedents of poor performance (Smallwood and Schooler, 2015). However, the thought sampling protocol is constrained by introspective accuracy and may be tainted by extraneous factors (e.g., Polychroni et al., 2024). Behavioral markers predicting attentional lapses may serve as an elegant method to study its repercussions. One marker is perceptual decoupling—a reduction of sensory processing from one's external environment. For instance, stimulus-evoked event-related potentials (ERPs) can be reduced during errors and prior to TUT reports, suggesting that these behavioral and subjective markers of lapsing attention represent similar cognitive states because they coincide with similar patterns of brain activity (Smallwood et al., 2008a). Perceptual decoupling has been further explored, revealing that periods of mind-wandering co-occur with a broader decrease in processing for all external stimuli regardless of relevance (Barron et al., 2011) and spontaneous changes in pupil size (Smallwood et al., 2011). Decoupling explains many of the consequences of absorptive mind-wandering on learning (Szpunar et al., 2013) and task performance (Baird et al., 2014). If perceptual input is compromised, then information is not available in one's mental workspace to encode and manipulate. This implies that a critical consequence of TUTs is that cognitive processes responsible for encoding and manipulating internal representations (e.g., working memory) may be impaired due to transient perceptual decoupling during CPTs.

**Figure 1:**
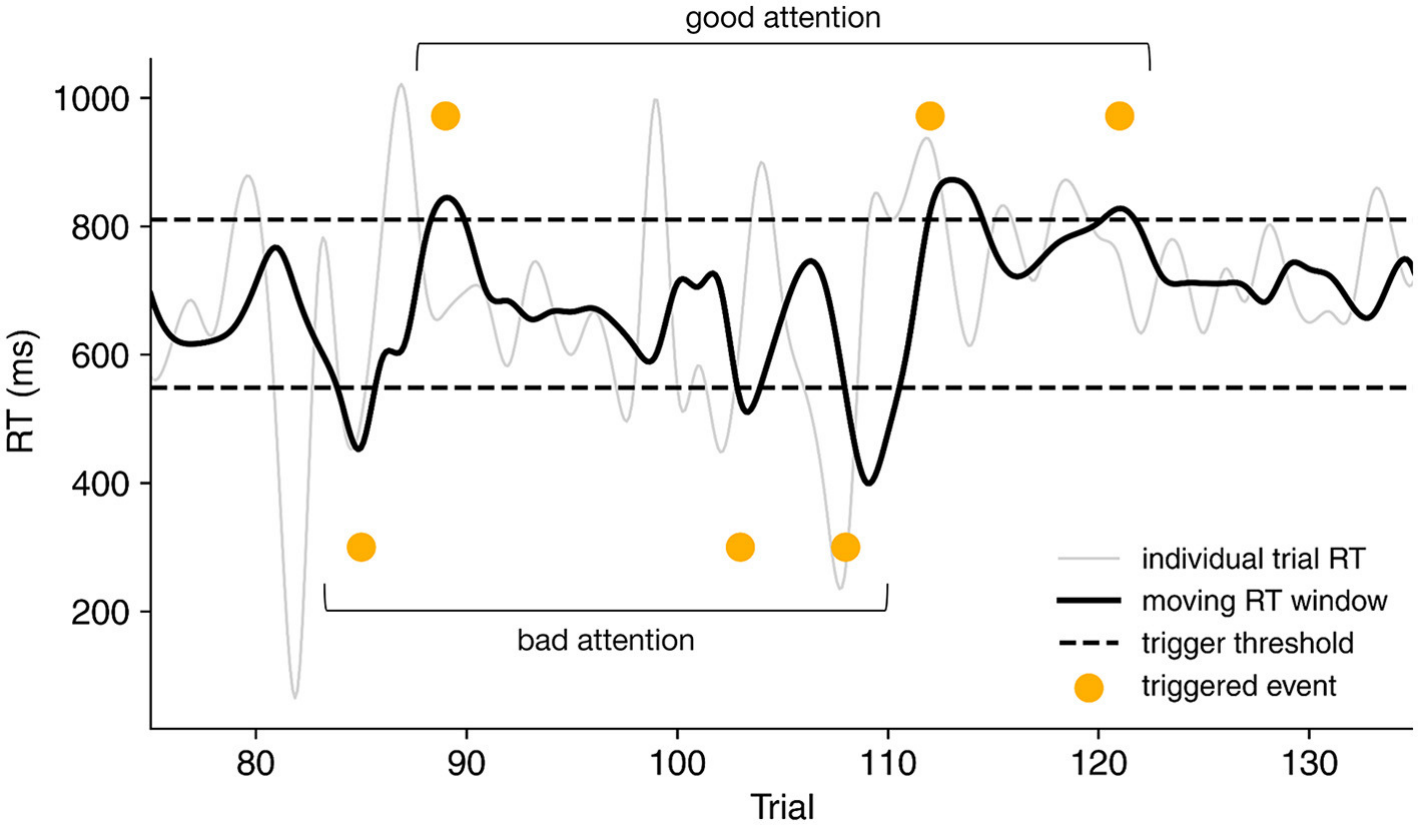
A schematic of a subject's individual trial RT, moving RT window, fast/slow thresholds, and triggered events in a real-time triggering procedure. The smoothed black line represents the three-trial moving RT average and the light gray line represents unique-trial RTs. When the three-trial window exceeds the slow or fast thresholds (dotted lines), an event is triggered, represented here as a gold dot. Slow-triggered events are considered “good” moments of attention, while fast-triggered events are considered “bad” moments.

Consistent with the conjecture that transient perceptual decoupling impairs processes responsible for storing and manipulating internal representations, the next studies that we review define lapsing attention as impaired memory encoding. DeBettencourt et al. (2019) used a paradigm that interleaved a SART with a working memory task. For the SART aspect of the paradigm, subjects pressed one key if an array of shapes were all squares and another key if they were all circles. One shape appeared more frequently than the other, promoting habitual responding to the frequent shape. Critically, each shape in a given array was a unique color. For the working memory aspect of the paradigm, subjects responded to occasional triggered events where subjects saw 3x3 arrays of colors at each shape location and clicked the color of the shape that had previously been in that location in the prior SART trial. The authors related sustained attention performance on the SART with accuracy on the working memory report using a RT-based triggering procedure to provide evidence that attention and working memory lapse together. Their triggering procedure, which used RTs from the SART, initialized a cumulative RT per subject in the first 10% trials, then defined ±1 RT standard deviation as upper and lower thresholds to determine especially fast or slow responding periods. The cumulative RT mean and SD were updated from trial-to-trial, and a three-trial RT window average was compared to the slow/fast thresholds to trigger the presentation of the working memory probes (e.g., Figure 1). Periods of especially fast responding preceded poorer performance on working memory trials compared to slow responding, and this was independent of any errors made in the continuous performance aspect of the task. The authors argued that this finding reinforced a causal effect of attention on working memory encoding, which aligns with predictions made based on perceptual decoupling during mind-wandering episodes.

In a second study, the triggering procedure was used to examine incidental long-term memory encoding as a function of fast or slow responding on a CPT (DeBettencourt et al., 2018). Subjects classified images in a continuous stream as either indoor or outdoor, with one of the categories being more frequent than the other. Infrequent category scenes required a different keypress and were once again triggered by three-trial RT means being ±1 SD from a subject's cumulative mean. The adaptive triggering design predicted poorer memory performance during a surprise recognition test after the CPT, such that fast responding predicted worse memory encoding when compared to slow responding. This again suggests that attentional state can be inferred based on RT speed, and that this can help predict future decrements in performance. Unlike their previous study with working memory probes, triggered events were always infrequent trials. Since fast triggers are more likely to catch errors, this raises the question of error-related processing explaining degraded encoding rather than lapsing attention (Seli et al., 2013). However, this was ruled out by a control analysis limited to correct infrequent trials showing the same pattern: fast responding preceded scenes that were not recognized in the memory posttest.

When considered together, these studies show that the real-time triggering procedure can effectively capture periods of good and bad attention without requiring *post-hoc* analyses to characterize attentional fluctuations within a subject. Additionally, three out of four of these studies showed that a separate cognitive task could be integrated into CPTs with triggering procedures to determine the role of vigilance across domains.

## 4 Discussion

A number of studies in the literature have attempted to identify patterns of RTs in CPTs that provide insight into continuous fluctuations in attentional state. The goal is that these patterns could serve as behavioral markers affording unobtrusive tracking and prediction of lapsing attention and task-unrelated thinking. In this review, we discussed papers that attempted to isolate such RT markers using two different approaches: *post-hoc* PCA and real-time closed-loop triggering procedures. In studies using the PCA approach, one recurring pattern was “speeding,” such that accelerating RTs preceded both errors and the subjective experience of mind-wandering in a CPT. However, the stability of this pattern as a marker of attentional state is somewhat unclear because replication attempts using larger samples and refined methodologies were unsuccessful. In contrast, the studies reviewed here using the real-time closed-loop procedures showed that RT speed of successive CPT trials provide an in-the-moment comparison between good and bad attentional states.

The closed-loop, real-time triggering approach has two key advantages. First, it is an adaptive, within-subjects approach. Second, closed-loop methods can be deployed in-the-moment and the studies that we reviewed here clearly demonstrate that real-time triggering is a viable alternative approach to detecting fluctuations in attentional state. This stands in contrast to previous work that often depends on *post-hoc* analyses on CPTs to identify periods where a subject is “in the zone” or “zoning out” to test interactions between attentional state and other phenomena (Wakeland-Hart et al., 2022; Decker et al., 2023a,b). Using the triggering method, DeBettencourt et al. (2018, 2019) demonstrated a relationship between lapsing attention and impaired memory encoding. The triggering procedure can be used to assess other issues; for example, researchers have addressed the role of attention in statistical learning, or an unconscious extraction of patterns in one's environment that largely relies on implicit memory systems (Turk-Browne et al., 2005). In the mind-wandering literature, there exists conflicting evidence as to whether implicit learning relies on attentional resources (Franklin et al., 2016; Brosowsky et al., 2021), but Zhang and Rosenberg (2024) used a triggering procedure to elucidate the nuanced interaction between statistical learning and attentional quality.

A critical next step is to test the sensitivity of triggering tasks to individual differences (Smilek et al., 2010) such as impulsivity (Helton, 2009), inhibitory capacity (Evans and Rothbart, 2007), and dispositional mind-wandering (Carriere et al., 2013; Mrazek et al., 2013). Doing so will shed light on the suitability of the custom triggering procedure in capturing lapses within a diverse sample. Encouragingly, DeBettencourt et al. (2019) found that lapses equally impaired memory encoding regardless of individual working memory capacity, suggesting that the method can be used to generalize the consequences of poor attention across populations with different cognitive capabilities. It is also possible that patterns of fast or slow responding may represent different cognitive states for different people.

Another exciting future direction includes coupling the RT triggering with physiological indices such as pupil size (Keene et al., 2022) or measures of brain activity (Kam et al., 2022; Dong et al., 2021; Fortenbaugh et al., 2018) to improve lapse prediction. For example, Groot et al. (2021) triggered the insertion of thought probes in real time based on RT while simultaneously measuring pupillometry, EEG, and fMRI to characterize biological markers that co-occur with task-unrelated thoughts and variable response patterns. Integrating other measures may support control analyses intended to rule out alternative explanations for errors on CPTs (Cheyne et al., 2009). Notably, using some of these other lapse-prediction methods may better map onto real-world scenarios such as driving (Lin et al., 2013) and reading (Hutt et al., 2024, Mills et al., 2021; D'Mello and Mills, 2021; Kuvar et al., 2023). RT-based methods are constrained by the requirement that they are integrated into monotonous CPTs. However, we argue that future theories and applications in sustained attention still may be enhanced through the integration of real-time triggering designs. For example, the study of the vigilance decrement, an observed decline in performance over time (Thomson et al., 2015; Warm et al., 2008), can be significantly advanced through this method because the task dynamically responds to fluctuations in RT. Experimental results might therefore offer a nuanced perspective on the temporal dynamics of attention. The severity of perceptual decoupling may steadily increase alongside the vigilance decrement, but may only be captured by speeding RTs during transient periods of poor control.

The real-time triggering approach allows researchers to not only detect when attention starts to wane, but also when to intervene, potentially mitigating the deleterious effects of lapses (e.g., Roberts et al., 2024). By setting thresholds for RTs that trigger cognitive or behavioral prompts, the efficacy of various interventions aimed at restoring attentional focus can be tested directly (Al-Shargie et al., 2019). The method offers a dynamic and responsive framework to study and counteract attentional failures, assaying high-stakes environments—such as driving, security monitoring, or medical screening—where lapses can lead to catastrophe.
